# Papillomaviruses: Viral evolution, cancer and evolutionary medicine

**DOI:** 10.1093/emph/eov003

**Published:** 2015-01-28

**Authors:** Ignacio G. Bravo, Marta Félez-Sánchez

**Affiliations:** ^1^Infections and Cancer Laboratory, Catalan Institute of Oncology (ICO), Barcelona, Spain; ^2^Bellvitge Institute of Biomedical Research (IDIBELL), Barcelona, Spain

**Keywords:** viral oncology, virus evolution, infection and cancer, molecular epidemiology, HPV, virus ecology cancer, vaccination, screening

## Abstract

Papillomaviruses (PVs) are a numerous family of small dsDNA viruses infecting virtually all mammals. PVs cause infections without triggering a strong immune response, and natural infection provides only limited protection against reinfection. Most PVs are part and parcel of the skin microbiota. In some cases, infections by certain PVs take diverse clinical presentations from highly productive self-limited warts to invasive cancers. We propose PVs as an excellent model system to study the evolutionary interactions between the immune system and pathogens causing chronic infections: genotypically, PVs are very diverse, with hundreds of different genotypes infecting skin and mucosa; phenotypically, they display extremely broad gradients and trade-offs between key phenotypic traits, namely productivity, immunogenicity, prevalence, oncogenicity and clinical presentation. Public health interventions have been launched to decrease the burden of PV-associated cancers, including massive vaccination against the most oncogenic human PVs, as well as systematic screening for PV chronic anogenital infections. Anti-PVs vaccines elicit protection against infection, induce cross-protection against closely related viruses and result in herd immunity. However, our knowledge on the ecological and intrapatient dynamics of PV infections remains fragmentary. We still need to understand how the novel anthropogenic selection pressures posed by vaccination and screening will affect viral circulation and epidemiology. We present here an overview of PV evolution and the connection between PV genotypes and the phenotypic, clinical manifestations of the diseases they cause. This differential link between viral evolution and the gradient cancer-warts-asymptomatic infections makes PVs a privileged playground for evolutionary medicine research.

## INTRODUCTION

Papillomaviridae are a diverse family of small, non-encapsulated viruses that infect warm-blooded vertebrates. Members of this family were initially described in mammals, but they have also been found in birds, turtles and snakes and probably infect all amniotes [1]. To date, more than 200 genotypes of distantly related human papillomaviruses (PVs) have been identified. In other well-sampled species, such as horse, dog, cow or cat, many distantly related PVs have also been detected. Further, virtually all humans are simultaneously colonized by several PVs, causing asymptomatic infections in skin and mucosa. Most likely, this is also the case for all other mammals. Thus, PVs are a fundamental part of the mammalian healthy skin microbiota. In most individuals, PV infections are acquired early during childhood and persist asymptomatically during all their lifetime. However, certain PV infections can have clinical presentations from self-limited benign growth, e.g. hand or plantar warts, to malignant growth, e.g. cervical or anal cancer. Indeed, cancers associated to chronic infections by a few oncogenic PVs are a major public health concern. Large screening programs for early detection of gynaecological chronic infections by oncogenic PVs were launched decades ago and are being complemented by systematic vaccination programs in the last years. However, our knowledge about PV molecular biology and natural history of the infection is deeper than our comprehension of the viral-host evolutionary and ecological interactions. We still need to understand how these novel anthropogenic selection pressures imposed onto a few PVs will affect both short-term and large-term dynamics between PVs and humans. This article aims to bridge the current gap between mechanistic and clinical research on the one hand and evolutionary and ecological research on the other hand, for PVs and the associated infections and diseases.

## BASICS ON PV BIOLOGY

Infection by PVs targets undifferentiated keratinocytes in the basal layer of the stratified squamous epithelia, at both cutaneous and mucosal levels. Most of our knowledge about PVs focuses on a handful of medically important, closely related human PVs (HPVs), linked to the development of anogenital and oropharyngeal cancers.

### PV genome structure

**Figure 1.**
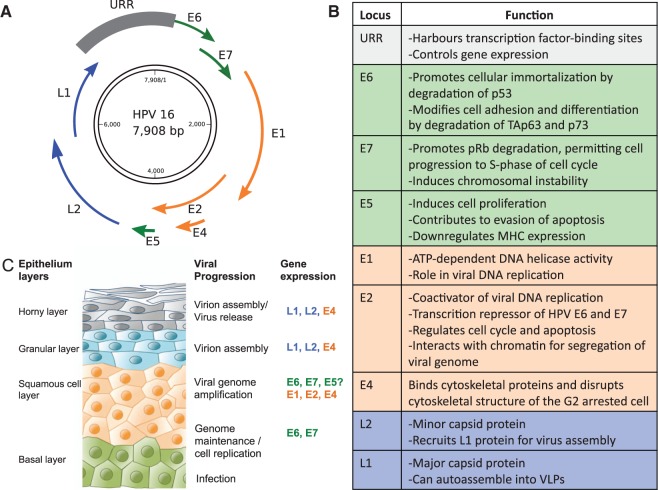
Genome organization and life cycle of PVs. (**A**) Schematic representation of PV dsDNA genome, exemplified on HPV16, showing the location of the early (E) and late genes (L) and of the URR. (**B**) Summary of PV genes functions. Genes involved in similar functions are indicated with similar colours: green, genes implicated in oncogenesis; orange, viral replication genes and blue, viral capsid genes. (**C**) Schematic view of the PV16 life cycle. The squamous epithelium is represented on the left and the genes expressed in each stage of the keratinocyte differentiation program are noted at the right. PVs infect keratinocytes in the basal layer of the epithelium that become exposed through microwounds. The viral genomes are established in the nucleus as low-copy episomes and early viral genes are expressed. The viral genomes are replicated in synchrony with cellular DNA replication. After cell division, one daughter cell migrates away from the basal layer and undergoes differentiation. Differentiation of HPV-positive cells induces the productive phase of the viral life cycle, which requires cellular DNA synthesis machinery. The expression of E6 and E7 deregulates cell cycle control, pushing differentiating cells into S phase, allowing viral genome amplification in cells that normally would have exited the cell cycle. The late-phase L1 and L2 proteins encapsidate newly synthesized viral genomes and virions are shed from the uppermost layers of the epithelium

PVs contain a circular double-stranded DNA genome of approximately 8 kb (Fig. 1), organized into three major regions: (i) an upstream regulatory region (URR) harbouring transcription factor-binding sites and controlling gene expression; (ii) an early region, encoding for six genes involved in multiple functions including viral replication and cell transformation and (iii) a late region, encoding for the L1 and L2 capsid proteins which self-assemble to yield the virion. The conserved elements shared by all PV members are the presence of an URR, the early proteins E1 and E2 (and possibly the E4 gene nested into E2) and the late proteins L1 and L2 [2]. Theoretically, these four proteins alone might fulfil the basic tasks of replicating, regulating, stabilizing and packaging of the viral DNA, eventually leading to the release of the virion progeny [3].

### PV life cycle

The best-studied PV is HPV16, a mucosotropic PV that is the primary cause of cervical cancer and of other anogenital cancers [4]. The life cycle described below corresponds to HPV16, and although it may be applicable to all PVs, differences in strategies leading to productive/silent, chronic/acute infections have evolved and may vary between different PV groups.

The infectious cycle of PVs is linked to the differentiation program of the keratinocyte (Fig. 1). The virion enters basal keratinocytes, probably targeting stem cells through microwounds or the hair follicles [5-7]. The precise nature of the cell surface receptor/s that allow for the initial attachment to the cell remains disputed [8, 9]. However, cell attachment and entry are not the limiting factors for infection, as they do not grant virus replication and virion production [10]. Actually, infection does not require the virus to be in form of virion, as skin abrasion and exposure to the naked viral genome are able to recapitulate the complete natural history of the infection in different animal models [11]. The naked viral genome is incorporated into the nucleus after the cell completes one mitosis cycle and replicates there as low-copy episomes. Genome integrity and correct segregation to the daughter cells is ensured by the E1 and E2 viral proteins [12, 13]. Viral replication is performed by high-fidelity cellular polymerases, in parallel to the replication of the cellular genome [14].

As keratinocytes migrate upwards and enter the differentiation process, they stop replicating and undergo changes in lipid biochemistry, protein specializations and fusion into cornified sheets for preventing water loss and eventually nucleus loss, cell death and shedding. The E6 and E7 PV proteins hijack the checkpoint mechanisms ensuring that the different cell cycle steps are completed properly. That allows the differentiating keratinocyte to enter uncontrolled proliferation [15, 16]. The E7 protein binds to Retinoblastoma family members and promotes their degradation [17], which results in the release and activation of the E2F transcription factor family, inducing unscheduled re-entry into S-phase cell cycle. The E6 protein prevents the induction of apoptosis in response to such unscheduled S-phase entry through degradation of p53 [16]. Finally, the E5 protein promotes hyperproliferation and prevents apoptosis of infected cells and is likely to facilitate malignant progression [18]. Thus, a chronic PV infection results in an increased proliferation activity in a cell that should not be replicating and is further depleted of quality control mechanisms. As a consequence of this unchecked cell cycle, the host cell accumulates mutations over time predisposing to the development of PV-associated cancers.

Following cellular differentiation, the expression of *E6* and *E7* is replaced by expression of *E1*, *E2*, *E4* and *E5* [19, 20]. As a result, viral copy number amplifies to thousands of copies per cell [21]. In the upper layers of the epithelium, viral gene expression shifts towards the L2 and L1 capsid proteins, which are targeted to the nucleus and autoassemble into virions, encapsidating the viral genome [22]. Finally, viral release proceeds without cell lysis. The E4 protein may contribute to viral egress in the upper epithelial layer by binding keratin filaments and disrupting their structure, but virions are essentially released through the normal process of desquamation [14].

### Clinical presentations of PV infections

The clinical manifestations of PV infection depend on multiple factors including the viral genotype, the genotype of the host, the type of epithelium infected (which could be considered as the phenotype of the host cell) and environmental factors such as the status of host immunity and nutritional factors. A detailed description of the different clinical presentations of PV-related diseases, for different hosts and anatomical locations is given in Supplementary Table S1.

The majority of PV infections are subclinical and do not cause any physical lesions [23]. PVs are commonly present in normal skin and mucosa of healthy individuals, suggesting commensalism/mutualism between PVs and their host cells [23, 24]. Infection by cutaneous PVs occurs rapidly after birth, as both viral DNA and antibodies are detected in infants and children [25, 26]. For certain PVs, the infection becomes clinical and may cause benign, self-limiting proliferative lesions, typically seen as warts affecting children in boundary epithelia in the fingers, lips and eyelids. Some lesions may be difficult to eradicate, evading immune surveillance of the host for a prolonged period of time. Nevertheless, in most cases, they are eventually controlled by the immune system and disappear after 2–3 years [27]. Sexual transmission of certain PVs is also recognized as a cause of anogenital warts, possibly the most common sexually transmitted disease [28]. However, not all PVs causing anogenital infections are sexually transmitted, as viral DNA and antibodies can be found in children and in individuals who had never had sexual intercourse [29, 30]. Anogenital PVs may be transmitted from mother to child by direct contact during labour [31, 32], and vertical transmission is often related to juvenile recurrent respiratory papillomatosis [33] and to genital warts [34]. However, the non-concordance of type specific PV between mother and newborn suggests the importance of additional horizontal transmission routes such as manipulation with infected hands, bathing, towels or fomites [28, 35, 36].

**Figure 2.**
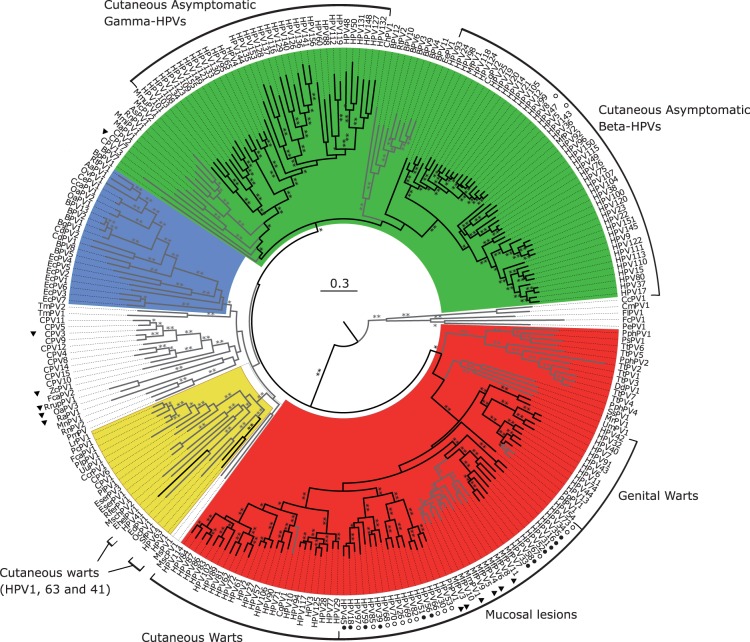
PV phylogeny reconstruction. Best-known maximum likelihood nucleotide phylogenetic tree of the concatenated *E1E2L1L2* gene sequences of full-length 263 PV genomes. Phylogenetic reconstructions yield four well-supported PV supertaxa. Colour code highlights the four crown groups: red, Alpha-Omikron-PVs; green, Beta-Xi-PVs; ochre, Lambda-Mu-PVs; blue, Delta-Zeta-PVs and white, PVs without well-supported phylogenetic relationships. Branches in black correspond to HPVs and branches in grey to non-HPVs. Outer labels indicate the most common tropism for the groups encompassing HPVs. Carcinogenicity of HPVs is indicated: a black dot indicates International Agency for Research on Cancer (IARC) group 1; a white dot indicates IARC groups 2A or 2B. Animal PVs with carcinogenic potential are marked with a black triangle. Asterisks on branches correspond to ML bootstrap support values. Two asterisks indicate maximal support values; one indicates support values between 90 and 50; and values under 50 are not shown

Only a limited number of evolutionarily related PVs cause persistent infections that pose a risk for development of high-grade lesions, which are precursors of anogenital cancers [37, 38]. Potential to induce malignant transformation is linked to specific activities of the E5, E6 and E7 oncogenes, exclusive to oncogenic PVs. Only the E6 protein in oncogenic PVs is able to induce degradation of the p53 cellular protein, thus promoting uncontrolled cell growth [39, 40]. Also, the E5 protein in oncogenic PVs decreases exposure of the infected cells to immune surveillance and decreases cellular dependence from external growth factors [41, 42]. Oncogenic PVs are responsible for virtually all cases of cervical and anal cancer cases and for a fraction of cancers of the penis, vagina, vulva and oropharynx (Fig. 2). Progression of precursor lesions to invasive cancer usually requires more than one decade, which allows time for the cancer screening programs, identification and treatment [27].

## PV DIVERSITY AND TAXONOMY

The study group of PVs, within the International Committee for the Taxonomy of Viruses, provides guidelines for PV classification and nomenclature (http://ictvonline.org/index.asp). Biological taxonomy is a human convention, and changing borders between categories reflect disagreements between splitters and lumpers. Virological taxonomy is also disputed [43], but a clear definition of viral taxonomical levels is essential for comparability between datasets. Since 2004, PV taxonomy relies on nucleotide sequence comparisons [44], and since 2010, PV classification should integrate phylogeny, genome organization, biology and pathogenicity [45]. The *L1* gene has been chosen as yardstick for building PV comparisons, and taxonomic categories are based on percentages of identity at the nucleotide level in this gene. Threshold definition is facilitated because the distribution of evolutionary distances among PVs shows a multimodal distribution [44–46], even if these distances are not homogeneous throughout the whole PV tree [46].

**Figure 3.**
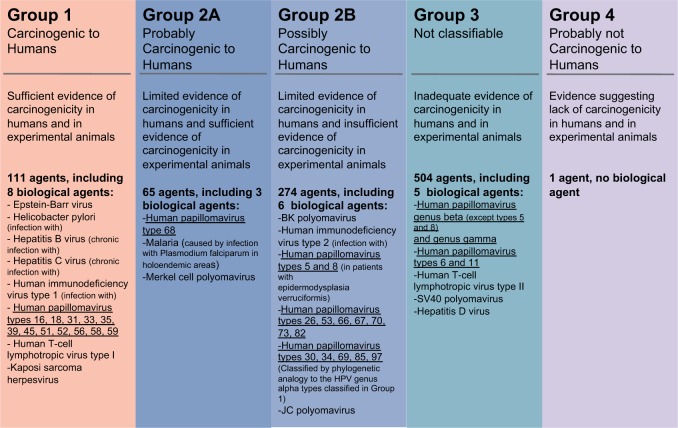
Classification of carcinogenicity used by the International Agency for Research on Cancer (IARC). IARC’s programme relies on international working groups of scientists expert in the particular area under investigation. The working groups analyze the information from case reports and epidemiological studies on humans, animal studies and other relevant biological data to evaluate the carcinogenicity of different agents to humans. Agents are classified into one of the four carcinogenicity groups. (Data extracted from IARC Monographs vol. 100B and 104.)

Viral taxonomy standards do not implement taxonomic categories below viral species. However, for the PV community, the clinically relevant taxonomic level is the ‘type’: two PV genomes sharing more than 90% nucleotide identity in the L1 gene belong into the same PV type [44]. Isolates from closely related PV types may show very different phenotypes. As an example, HPV16 and HPV31 are sister taxa and display similar tropism, but HPV16 is 15 times more prevalent in cervical cancer than HPV31 [47]. Similarly, HPV6 and HPV11 are sister taxa and cause similar productive lesions, but differ in tropism, as HPV6 is more often associated to genital warts, whereas HPV11 is more often associated to respiratory papillomatosis [48, 49]. The International Agency for the Research on Cancer classifies also PVs at level of type depending on their carcinogenicity (Fig. 3). The clinical focus at this taxonomic level is further obvious in the current developing trends of commercial assays for PV identification, essentially in the context of cervical cancer screening, which provide tools for genotyping always at the level of PV types [50], as well as in the choice for vaccination targets.

**Figure 4.**
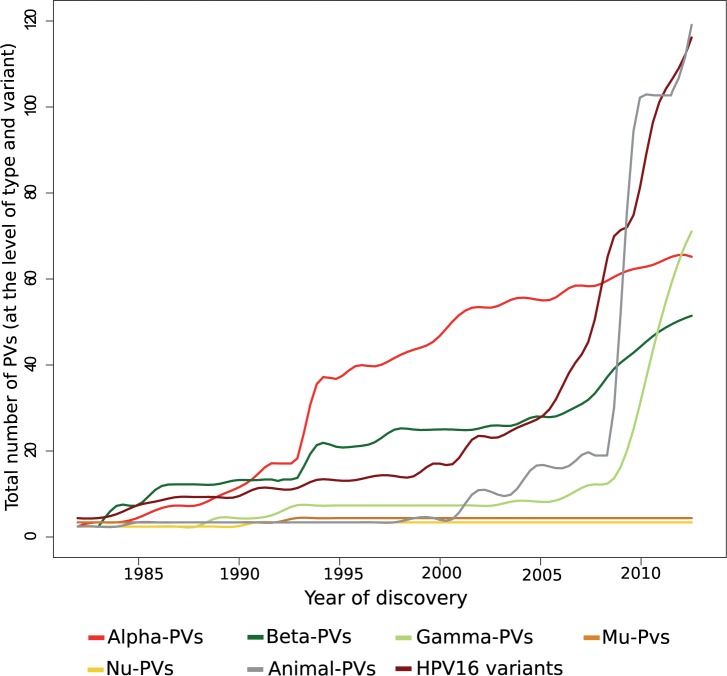
Evolution of the number of PV sequences available in the GenBank. Data have been extracted at the level of type for the genera encompassing PVs infecting humans: *Alpha-*, *Beta-*, *Gamma-*, *Mu-* and *NuPVs*. The evolution of the number of different full-length genome HPV16 variants is also shown. The trends in the number of sequences available indicate that although the members of *AlphaPVs*, the best-studied PVs, have reached a plateau, the number of *Beta-* and *GammaPVs* is still increasing. Only three members within *Mu-* and *NuPVs* have been described. Although largely undersampled and constantly growing, the number of animal PVs represents one-third of the global known PV diversity

Around two-thirds of the full-length genome PV types entries in the Genbank correspond to HPVs. The strong research focus on PVs and cancer has led to the description of around 60 types in the *AlphaPVs* genus, which harbours all oncogenic HPVs associated to anogenital cancers. The advent of rolling circle amplification first and of next-generation sequencing later have largely expanded the number of HPVs classified as *Beta*- or *GammaPVs*, with both genera spanning now over hundred HPV types. Results from metagenomic surveys suggest that we may have already identified most human *AlphaPVs* [51], whereas most of the unexplored HPV diversity may belong within the *Beta*- and *GammaPVs* [52, 53]. This trend is also reflected in the evolution of the number of PV sequences available in the Genbank, with *AlphaPVs* reaching a plateau, whereas *BetaPVs* steadily increase and the number of *GammaPVs* has rocketed (Fig. 4). It is very interesting to note that two PV genera (*Mu*- and *NuPVs*) encompass only three HPVs for which no close relative has been described thus far. Should this difference in number be true, it would imply a large variation in differential success, as measured in terms of number of lineages infecting the same host, for different PV genera, in the sequence *GammaPVs > BetaPVs ≥ AlphaPVs >> MuPVs ≥ NuPVs*.

## PV EVOLUTION

The consensus in the PV community poses that partial phylogeny mirroring between hosts and viruses suggests that virus–host coevolution is the main driving factor of PV evolution, even if other mechanisms also contribute significantly [54, 55]. PVs are thus conceived to be well adapted to their hosts and to evolve slowly.

### Mutation and substitution rates in PVs

PVs do not encode for a DNA polymerase, and the viral genome is replicated during S-phase by high-fidelity cellular polymerases with error correction. Mutation rates and mutational biases ought to be thence close to those of the hosts. However, genome composition and codon usage preferences in HPVs do not match those of the host genome. They are instead enriched in A+T and display extreme codon usage preferences [56–58], as much as that there is not a single instance of match between the most used synonymous codon by HPVs and by human genes [56]. These compositional differences possibly reflect a bias in the mutation/selection evolutionary processes that still needs to be understood. Regarding mutation, it is conceivable that viral infection could modify the polymerase biases by altering the biochemical environment for PV genome replication. Additionally, the PV genome could be a target for the cellular APOBEC3 cytidine deaminase [59], resulting in a C>>T bias similar to those observed in several cancer genomes [60]. Finally, viral genome replication occurring in superficial layers of the skin may be exposed to increased UV radiation and therefore subject to additional error-prone mechanisms linked to resolution and repair of cyclobutane pyrimidine dimers [61]. Regarding selection, three adaptive explanations for the biased codon usage preferences in PV genes have been proposed. First, wild-type PV genes are usually poorly translated in cell culture [62, 63]. It has been classically claimed that PV codon usage preferences have been selected for because they decrease viral protein synthesis, thereby lowering immune exposure [64] and experimental evidence *in vivo* with the rabbit model also points in this direction [65]. Second, it has been postulated that codon usage preferences in PVs may have evolved to match the varying tRNA profile of the keratinocyte through the differentiation program [66]. Finally, the barely 8-kb PV genome accommodates overlapping genes, transcription, regulatory and splice sites [67] and is subject to complex differential methylation during the life cycle while avoiding accumulation of CpG islands that could elicit immune response [68]. The biased codon usage preferences of PV genes may thus reflect the trade-off between all these forces optimizing the protein coding and non-protein coding information encrypted on the viral genome.

Estimates for PV substitution rate, i.e. the rate at which mutations are fixed in the PV genome, fit well our common understanding of viral mutation rates, with dsDNA viruses showing the slowest evolutionary rates among viruses [69, 70]. Estimates for PV coding regions render values between 2 × 10^−8^ and 5 × 10^−^^9^ substitutions per site per year [71, 72], whereas the non-coding, regulatory region of the PV genome accumulates mutations around two-times faster than the coding regions [73, 74]. These values are slightly higher but around the same order of magnitude than for mammals, reinforcing the idea that PVs use the cellular polymerases with proofreading capacity. Experimentally, the PV episome can be maintained in cell culture at numbers of 500 copies per cell without obvious generation of diversity among the intracellular viral genomes, as no variation has been reported with a frequency above 0.5% in the W12 cell line, which harbours HPV16 episomes [75]. Thus, evolutionary rates in PVs may occur too slowly to be studied by means of serial sampling or historical clinical material. Analyses of HPV16 sequences show that there is not enough signal-over-noise to infer evolutionary rates by means of root-to-tip phylogenetic regression [76]. Indeed, two BPV1 isolates sampled in Sweden and in Wisconsin more than 30 years apart displayed 99.89% nucleotide identity, not different from the standing genetic variation of this virus [77].

### Recombination in PVs

The common understanding about PV dynamics is that recombination plays a minor role in PV evolution. However, the molecular evidence suggests that recombination is central to PV genome replication, and phylogeny and comparative genomics pinpoint several recombination events along the evolutionary history of PVs. Some of these events have had profound implications for colonization of new niches and the emergence of oncogenic phenotypes.

PV replication requires homologous recombination activity. Replication of the PV genome occurs bidirectionally during the non-productive stages of the infection, yielding episomes [78] and switches during the productive stages of the infection towards a rolling circle-like replication that generates concatenated viral genomes [79]. Homologous recombination may provide the molecular tool for resolving, excising and re-circularizing the concatenated genomes into individual plasmid genomes that are eventually packed as virions [80]. Resorting to homologous recombination might allow for rare error events of non-homologous recombination and indeed, the presence of recombinant HPV16 sequences has been reported during natural infection [81]. Using the rabbit model, experimental infection with a mixture of complementing, truncated viral genomes resulted in productive lesions that contained possible recombinant sequences from both parental DNA sequences [82].

**Figure 5.**
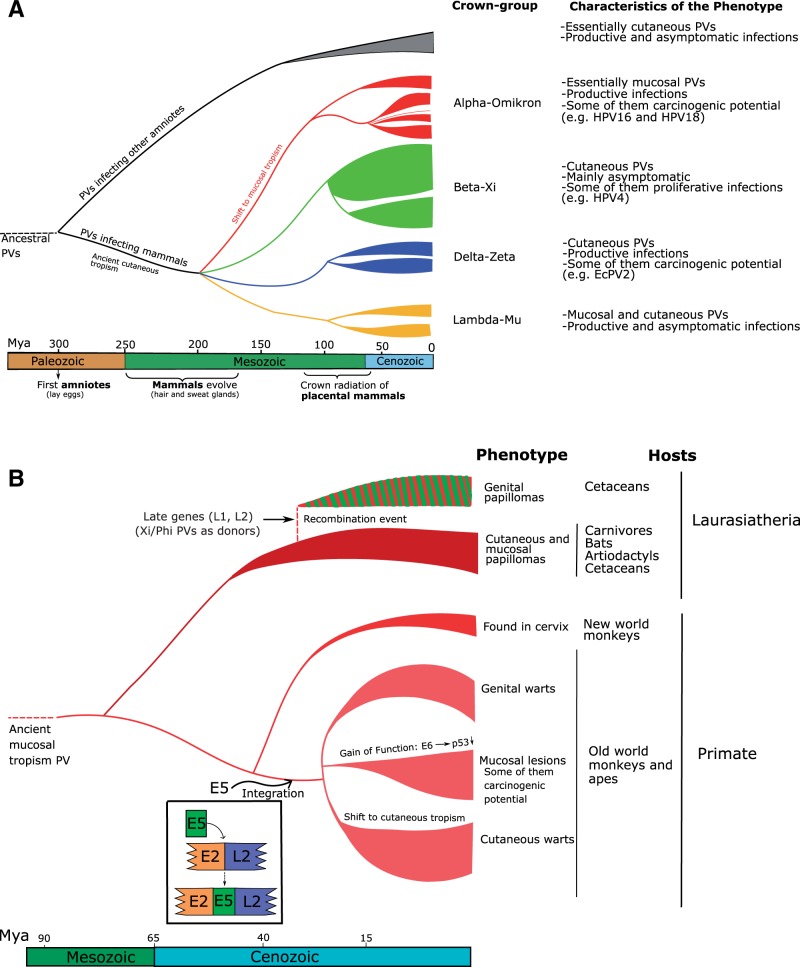
Global scenario of PV evolution. (**A**) Ancestral amniotes were already infected by ancestral PVs. The four PV crown groups (labelled in red, green, blue and orange) appeared during the evolution of skin glands and hairs (250–150 Mya). Subsequent mammalian radiation triggered further a second wave of PV diversification (110-60 Mya). (**B**) Zoom into the evolutionary scenario for lineages in the Alpha-Omicron-PVs crown group, with individual, rare events largely influencing the evolutionary history. A recombination event yielded a novel viral lineage with the early genes from an Alpha-Omicron-PV infecting cetaceans and the late genes from a Beta-Xi-PV infecting artiodactyls. Separately, in a PV lineage infecting the ancestor of Old World monkeys and apes, an integration event between the *E2* and the *L2* genes introduced a DNA segment encoding for the ancestral E5 ORFs. This integration triggered an adaptive radiation that generated three viral lineages with different tropism and different clinical manifestations. In one of these lineages, the E6 proteins acquired the ability to degrade p53. Some viruses in this lineage are responsible for anogenital and oropharyngeal cancers in humans

Several independent recombination events have also shaped the evolution of Papillomaviridae. The clinically important *AlphaPVs* have undergone recombination event(s) between the early and the late regions of the genome [42, 83, 84]. As a consequence, the phylogenetic relationships among *AlphaPVs* differ when inferred based on the early or the late genes. Reconstructions based on genes related with transformation or with replication (i.e. early genes) cluster together viruses associated with similar clinical manifestations, cutaneous warts, benign mucosal proliferative lesions or mucosal lesions with malignant potential. This pattern disappears when the phylogeny is based on the capsid genes (i.e. late genes) [42, 83]. This recombination event is most likely related to the integration of the ancestral *E5* ORF on the backbone of the ancestral *AlphaPV* genome, as there is a clear match between the E5 genotype and the associated phenotype of the infection [42]. The integration of the *E5* gene provided a way to immune evasion by modifying membrane chemistry [85, 86] and decreasing presentation of viral epitopes [87, 88]. Hence, the acquisition of a novel repertoire of mechanisms for sustaining cell growth and for evading immune response triggered an adaptive radiation that generated the three main lineages of the *AlphaPVs* [42]. Integration of this E5 block occurred in the boundary between the early and the late regions of the PV genome backbone (Fig. 5). This genomic locus has been implicated in at least five independent integration events during the evolution of the Papillomaviridae [2, 89], either involving additional coding regions, such as E5 ORFs in *DeltaPVs* or in *TauPVs*, or long non-coding regions of unknown function in different members of the Lambda-MuPV crowngroup and of the Beta-XiPV crowngroup.

Recombination events have also occurred between distantly related PVs. This has been the case of certain patchworked monophyletic PVs infecting cetaceans, sharing the early genomic region with cetacean PVs in the Alpha-OmicronPV crowngroup and the late region with bovine PVs in the Beta-XiPV crowngroup [55, 90, 91]. Finally, recombination between very distant viral relatives can also lead to fixation, as in the chimeric viruses retrieved from bandicoots and displaying genomic features of two different viral families: the early, transforming genes of polyomaviruses and the late, capsid genes of PVs [92]. Both Papillomaviridae and Polyomaviridae are small circular dsDNA viruses, and the convergence on similar replication mechanisms may allow for such extremely rare events of non-homologous recombination. Fixation of non-homologous recombination shows a preference towards events involving non-coding regions, possibly because the probability of a non-homologous recombination event involving a coding region to maintain gene functionality is very low.

Globally, recombination has played a role during the evolution of Papillomaviridae, as it has been shown to occur *in vivo*, and several independent instances of fixation have been documented at very different levels of the PV tree.

### Within-host evolution

The study of within-host evolution is key for understanding the evolutionary dynamics of rapidly evolving viruses, such as human immunodeficiency virus or hepatitis C virus [93, 94]. For PVs, because of the low evolutionary rate, it is commonly assumed that generation of viral diversity during the course of PV infection is negligible. However, given the combination of large population size, large infection time and large prevalence, the study of generation of diversity in chronic PV infections and its connection with the differential outcome of the disease deserves deeper attention.

A careful analysis of published data reveals that generation of diversity does occur during chronic PV infections. Retrospective sequence analysis of HPVs in recurrent respiratory papillomatosis showed no evidence of strain replacement in 67/70 cases during a median follow-up of 4 years, with an individual case of 22 years follow-up [95]. However, in 5% of the patients, the original strain was replaced by another one very closely related, which could be explained either by viral replacement or by intrapatient evolution [95]. Similar results have been reported from the study of PV infections in women in consecutive genital/cervical samples. In most cases of persistent infection with HPV16, the same variant is retrieved during serial sampling [96–98]. However, changes in the predominant variant, as identified by changes in one or two nucleotides through consensus sequencing, have been reported in 4-8% of women during a follow-up of up to 2 years [97, 99–101]. Retrieval of the same PV variant during consecutive samplings is usually interpreted as evidence for persistent infection, whereas retrieval of a different variant is interpreted as a novel infection and never as a result of a bottleneck or of a selective sweep upon mutation. Claiming the case of selective sweep requires evidence for generation of diversity during the course of an infection; indeed deep sequencing of viral genetic material from clinical lesions showed that 3/7 samples contained polymorphic sites above the reliable mutation threshold, reaching frequencies of up to 5% for the minor sequences [75]. The study of viral persistence has traditionally been assessed through Sanger sequencing or by amplicon hybridization with probes targeting variant-specific polymorphisms, but such approaches are unlikely to capture the dynamics of slowly evolving viruses.

Conspicuous evidence of PV intrapatient evolution has been described in two independent cases of lung cancer developed in patients after a 20-year long history of recurrent respiratory papillomatosis, associated to HPV6 [102] and to HPV11 [103]. In both cases, viral genomes retrieved from the malignant lesions contained duplications of the regulatory region, a feature absent from the viral genomic sequences retrieved from benign lesions in the same patient. Colonization of a novel niche—the lung epithelium in both cases—may thus have provided with an evolutionary advantage to a rare mutant that appeared after a recombination event resulting in duplication of the regulatory region. These examples of parallel intrapatient evolution illustrate that highly prevalent, long-lasting infections by PVs result in viral effective population size values large enough to efficiently explore sequence space and to allow mutants with an advantage to be fixed, even if mutation rate and if recombination rate are (very) low. Along this line, HPV6 and HPV11 are common in healthy tissue of the female anogenital tract [104] and appear as the main causative agents of genital warts [105] but are associated with occasional cases of anogenital carcinomas [106]. Characterizing the PV population in such rare malignant lesions may help understand the intrapatient viral dynamics in slow evolving viruses.

### Ecology of the virus–host interaction

Viruses causing acute infections usually transform the infected cell into a virus factory, eventually leading to cell death and release of the viral progeny, while eliciting a strong, protective immune response [107]. PVs, however, do not kill the infected cell. Instead, most PV infections persist for decades but are not very productive. Only warts caused by PVs have a faster clinical course and are highly productive lesions. Natural infection by PVs elicits specific immune response in most cases [26, 108] but only a limited number of individuals develop high antibody titres that provide some degree of protection against reinfection with the same type [109]. Further, infections by multiple PVs in healthy women and in low-grade lesions are more common than single infections [110]. Nevertheless, it is not clear whether certain PVs tend to appear together in co-infection patterns more often than expected by chance [111–113]. The presence of multiple infections by oncogenic PVs has an additive effect on the risk of developing high-grade lesions [114]. However, the mutual interactions between types causing simultaneous infections and the interplay with the host’s immune system are not necessarily correlated with the occasional development of cancer, because infectious tumours derive most likely from a single clonal expansion event [115] and individual lesions are associated to individual PVs [116].

Oncogenic potential in PVs is linked to a viral monophyletic lineage (Fig. 5a), characterized by two specific synapomorphies: the ability of the viral E6 protein to induce degradation of the cellular p53 protein [39, 40], and the presence of a particular E5 protein, able to downplay immune exposure in infected cells [42]. The combined effect of both viral activities allows these viruses to establish chronic infections through sustained low-level replication of the infected cell and immune escape. Such chronic infections produce very low amounts of virions but last for decades. The non-oncogenic sister lineages (Fig. 5b) have instead evolved towards benign lesions that grow fast and produce large amounts of virions but that are ultimately controlled by the immune system and cleared. A strong trade-off between virion productivity and immune exposure is thus evident in the alternative evolutionary strategies of sister viral lineages. Long-lasting uncontrolled cellular replication in chronic infections eventually leads to accumulation of mutations [117] and in some cases to genomic instability linked to integration of the viral genome in the cellular genome [118, 119]. Malignant growth is therefore a non-adaptive consequence of the increased potential in oncogenic PV for persistence without eliciting immune response, and cancers should be conceived as a sink in the ecological source and flux dynamics of PV infections. In the natural history of PV infections, cancers are a very particular stage because they are a double dead end: (i) for the virus, because cancers virtually do not produce virions and are therefore not infectious and (ii) for the host, because invasive cancers do not spontaneously revert, whereas precancerous lesions do spontaneously revert in the majority of cases following immune activation.

Studies on the time trends in HPV type prevalence in cervical cancer during the last 70 years have shown that the relative contributions of the different oncogenic HPVs have not varied from 1940 to 2007 [120]. Also, viral DNA similar to HPV18 and to HPV91 was retrieved from a genital lesion in a female XVI-century mummy, [121]. Nevertheless, stability of HPV type prevalence values in cancer does not necessarily imply stability of HPV type prevalence values in the healthy population, in the same way that viral prevalence in cancer [47] does not reflect circulating viral prevalence [104]. Globally, our ecological understanding of cancers linked to PV infections is still very poor, especially when compared with the strong epidemiological research developed around the burden of HPVs-related diseases.

### Long-term evolution of PVs

Coevolution with their hosts has been historically considered as the main force driving PVs evolution [122]. However, virus–host coevolution contributes to explain barely one-third of all events needed to reconcile the evolutionary histories of PVs and their hosts [55]. Other mechanisms such as intrahost duplication, lineage sorting—‘missing the boat’—or host-switch [123] need to be invoked to fully explain the global diversity of PVs and their relationship with their hosts [54, 55]. These results should, however, be interpreted with caution because PV hunting has been systematic in humans but remains opportunistic in most hosts, thus overestimating the contribution of intrahost duplication and of lineage sorting [1, 55]. Further, analyses of virus–host coevolution require knowledge on host-specificity that is commonly missing, and broad host-range may be more common than anticipated for PVs [24, 55, 89, 124, 125]. Finally, evolutionary relatedness between HPV16 and HPV18 sequences retrieved from the same geographical continent has served to sustain the claim for coevolution between PVs and human populations in recent times [74, 126], although this match was not observed for HPV6 [127]. From a more recent comprehensive sampling of the worldwide HPV16 diversity [128], we suggest that recent PV evolution may have been punctuated by episodes of expansion and bottlenecks/selective sweeps that deserve further study.

The evolutionary scenario that fits best the current description of the PV genetic diversity is a series of basal duplication events followed by limited virus–host coevolution [55] (Fig. 5). The *E6* and *E7* genes are very divergent, and the organization of these loci is highly variable across the PV tree. The ancestral PV, containing at least the core of the *E1-E2-L2-L1* genes, may have already infected ancestral amniotes some 300 Mya, by the time of divergence between the ancestors of birds and the ancestors of mammals [2]. During some 100–150 My, mammals evolved a glandular epithelium, associated to changes in beta-catenin pathways, lipid complexes and keratinized structures, that resulted in sebaceous, sweat and mammary glands and ultimately hairs [129]. Ancestral PVs may have diversified while colonizing these new niches, generating the ancestors of the extant PV crowngroups. No PVs have been retrieved—yet—from monotremes, but the presence of a PV pseudogene integrated in the platypus genome shows that they have been exposed to these viruses [130]. The single PV genome retrieved from a marsupial host is not basal to all PVs infecting placentals [131], suggesting that the initial PV diversification predated the split between both mammalian clades. The ancestral placentals were thus already infected by several ancestral PV lineages, and viruses expanded with their hosts as they radiated. The Alpha-OmicronPV crown group evolved towards an essentially mucosal tropism, whereas the Beta-XiPV crown group evolved towards a commensal cutaneous phenotype. The conspicuous absence in the Delta-ZetaPV crown group of PVs infecting primates suggests an event of lineage sorting. More recently, one recombination event involving the ancestral *AlphaPVs* occurred before the split between old world monkeys and apes and led to the integration of small hydrophobic ORFs with oncogenic activity, the future *E5* genes [42]. The novel genomic resources triggered an adaptive radiation that generated at least three lineages allowed for a change of tissue tropism and diversified the phenotypic presentations of the infection (Fig. 5, Supplementary Table S1). In one of these lineages, the *E6* protein evolved later a gain of function favouring degradation of p53 [39, 40]. All oncogenic PVs associated to human anogenital cancers stem from this latter lineage and share a recent common ancestor [42, 132], possibly contemporaneous with the split between old world monkeys and apes (Fig. 5).

At shallower levels, the evolutionary forces driving diversification and differential ecological success between closely related viruses, such as the successful HPV16 and the obscure HPV35, are not well known. Albeit not systematically, experimental interspecies transmission was explored since the early stages of PV discovery [133, 134], and cross-species infection occurs under natural conditions [55, 89, 124, 125]. For certain sister PVs infecting closely related hosts, the barrier to cross-species transmission might rather be cultural than biological. This may be the case of HPV13, PtPV1 and PpPV1, causing similar oral proliferative diseases in humans, chimpanzees and bonobos [135–137]. Such cultural barriers allowing for isolation and fixation of viral lineages may be especially effective when viral spread is linked to intimate or sexual host contact and when the viruses involved mutate slowly, as is the case for PVs or for certain herpes viruses [138, 139].

### Novel ecological pressures linked to vaccination

The introduction of vaccines targeting a subset of the circulating PV diversity implies a dramatic change in the differential ecological pressures to virus circulation. Therefore, evolutionary and ecological considerations on vaccines and PV dynamics have both fundamental and clinical implications. Such considerations address the individual levels of protective immunity elicited by vaccination, the possible generation of herd immunity, i.e. the protection against viral infection in non-vaccinated individuals elicited through barrier effect of vaccinated individuals and the possibility for the pathogen to evade immune restrictions through sequence evolution.

**Figure 6.**
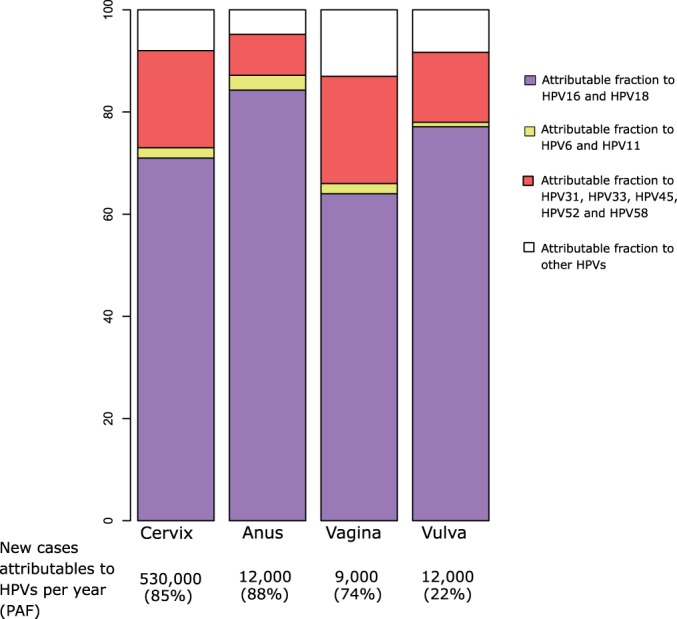
**Fraction of anogenital cancers caused by HPVs infections preventable through vaccination.** Data should be read as follows, with vaginal cancer as an example: every year, 9,000 new vaginal cancer cases are diagnosed worldwide, and 74% of them are associated to infection by HPVs. This is known as PAF, Population Attributable Fraction. From these, 77.1% are associated to HPV16 or HPV18, and could be prevented using the bivalent or the quadrivalent vaccine; 0.9% are associated to HPV6 or HPV11, and could be prevented by the quadrivalent vaccine; 13.7% of the rest of HPV-related cases are associated to HPV31, HPV33, HPV45, HPV52 and/or HPV58, and could be prevented using the nonavalent vaccine; the remaining 8.3% of the vaginal cancer cases are not targetted by any current vaccine. Data extracted from the Catalan Institute of Oncology HPV Information Center, last queried on June 2014 (http://www.hpvcentre.net/)

Two prophylactic HPV vaccines are currently available: a bivalent vaccine targeting HPV16 and HPV18 [140], and a quadrivalent vaccine additionally targeting HPV6 an HPV11 [141]. Both contain recombinant L1 proteins that autoassemble into hollow structures mimicking virions, called virus-like particles. An enhanced vaccine including virus-like particles from five additional targets—HPV31, 33, 45, 52 and 58—has just been licensed [142]. This vaccine is intended to prevent infection by HPVs responsible for the majority of anogenital cancers (Fig. 6), and it is envisioned that extending the repertoire of viruses in the formulation should suffice to cover eventual type replacement dynamics [143]. Despite their incomplete status, these pseudo-viral structures can elicit protective antibodies [144]. Indeed, immunization results in generation of high antibody titres in above 95% vaccinated individuals [140, 141]. Vaccination delivery of the viral antigens by intramuscular and the presence of adjuvant molecules acting as local immune modulators are possibly responsible for the very high level of seroconversion and for the high antibody titres compared with those elicited during natural infection [14]. Additionally, immunization results in partial cross protection against viruses not directly targeted by the vaccine formulation, essentially HPV31 and HPV45, close relatives of HPV16 and HPV18, respectively [145–147]. The strong immune response elicited through vaccination and the sexual transmission dynamics of infection predicts a strong herd immunity effect [148], compared with that induced by the limited immune response to natural infection [149]. Indeed, data on the decrease of incident cervical lesions and genital warts suggest that vaccination results in the establishment of a herd immunity effect in unvaccinated women and partly also in unvaccinated young men [146, 150, 151].

Pathogens targeted by vaccination may evolve escape mutants that render the vaccine ineffective. For PVs, such phenomenon is described in the literature as type replacement. The strong protective response against the targeted viruses, the induced cross protection and the low evolutionary rate of PVs have led to the consideration that type replacement after massive vaccination is unlikely [143, 152]. However, interactions between PVs and the immune system remain difficult to interpret. As an example, acquisition of a novel infection by HPV33 seems to occur more often in unvaccinated people already exposed to other oncogenic HPVs [153]. Nevertheless, a major threat for type replacement after vaccination would be the emergence of a viral recombinant encompassing the early region of one of the highly oncogenic PVs and the late region of one virus not targeted by the vaccine [1]. Even if both, recombination rate and the probability of such two viruses to simultaneously infect the same cell are very low, combination of large population size and chronic infection may provide the (remote) possibility for such an event to occur, and the selective advantage under the enormous pressure of the vaccine-mediated immunity would result in spread of the recombinant.

A few ecological models addressing the dynamics between anogenital PVs and humans have been developed. They have focused on the trade-off between virion production and immune exposure [154], on the connection between PV infection, cell differentiation and epidermal dynamics [155], on the intrinsically patchy nature of the PV infection in the epithelium and the competition between simultaneous viral infections and the immune system [156] and on the impact of acquisition and clearance of concurrent infections [157]. Although limited and simplified, results from these models are valuable as they suggest that many of the common assumptions regarding intrapatient and epidemiological dynamics of the PV-human interaction may need to be revised. Specifically, we still need to understand why natural immunity does not always generate protective immune responses [144, 149]. Such information is essential to make a choice between ‘susceptible-infected-resistant’ and ‘susceptible-infected-susceptible’ models [109]. We need to decipher whether the interactions between PVs are or not neutral [111–113] and how multiple infections influence the probability of clearance of each individual virus, because predictions on type replacement largely differ depending on whether sequential or simultaneous clearance is assumed [157]. Finally, integration of the viral mechanisms leading to local impairment of immune response [158–160], of the clonal nature of each PV lesion [115, 116] and of the complex composite structure of the target epithelium [161] will render modelling approaches closer to the complexity of the biological question [155, 156].

## CONCLUSION

Ever since mammals evolved skin glands and hairs, their epithelia and mucosa have been infected by a plethora of PVs. Decades of fundamental research have shown that PVs have a broad genotypic diversity, have experienced complex evolutionary histories, are capable of hijacking the cellular and immune systems at several levels and are associated to multiple manifestations of the infection, from asymptomatic to invasive cancer. Although infections by closely related PVs tend to display similar clinical presentations, the forces linking viral genotypic and phenotypic diversity with host/viral ecology have not been elucidated yet. We propose that PVs are an excellent model system for the study of chronic infections and the evolutionary interplay between innate and adaptive immune responses and that could become the reference model in evolutionary medicine for the study of cancers associated to infections. First, PVs offer a large repertoire of very divergent viral sequences, well sampled at several different evolutionary scales, suitable for in-depth evolutionary and phylogeographic analyses, which are still wanting. Further, PVs infections display large variation gradients in several key phenotypic traits, such as productivity, prevalence, immunogenicity, oncogenicity and clinical presentation. Several of such traits display opposite gradients and are clear examples of evolutionary trade-offs, chiefly between virion productivity and immune exposure. Such combination of genotypic and phenotypic diversity is unique among human pathogens. Finally, human interventions such as massive vaccination against selected PVs, as well as cancer screening focused on selected PVs, will surely have a positive impact on human health. However, their outcome on viral circulation, intrahost dynamics and epidemiology cannot be ignored, and we are still far from being able to foresee the associated impact. The combined efforts of epidemiology and ecology, both at the intrapatient and at the population level, will be required to understand the present and to anticipate the future of the long lasting interaction between the few oncogenic PVs and humans.

## SUPPLEMENTARY DATA

Supplementary data are available at *EMPH* online.

## FUNDING

This work was partially funded by the disappeared Spanish Ministry for Science and Innovation (CGL2010-16713). MF is the recipient of an IDIBELL PhD fellowship.

**Conflict of interest**: None declared.

## Supplementary Material

Supplementary Data
