# Supplementary material for: Papillomaviruses: Viral evolution, cancer and evolutionary medicine
Source: Evol Med Public Health. 2015 Jan 28;2015(1):32–51. doi: 10.1093/emph/eov003 (PMC4356112; doi:10.1093/emph/eov003)
Supplement: Supplementary Data [file supp_2015_1_32__index.html]

Papillomaviruses — Supplementary Data 

# Papillomaviruses

## Supplementary Data

files

**Files in this Data Supplement:**

- Supplementary Data - docx file
- Supplementary Data - xlsx file
