# Supplementary material for: Papillomaviruses: Viral evolution, cancer and evolutionary medicine
Source: Evol Med Public Health. 2015 Jan 28;2015(1):32–51. doi: 10.1093/emph/eov003 (PMC4356112; doi:10.1093/emph/eov003)
Supplement: Supplementary Data [file supp_eov003_New_Microsoft_Office_Word_Document.docx]

**Table S1: Clinical presentation of genomically characterized papillomaviruses**. Colour code highlights the four crown groups: red, Alpha-Omikron-PVs; green, Beta-Xi-PVs; ochre, Lambda-Mu-PVs; blue, Delta-Zeta-PVs; and grey, PVs without well-supported phylogenetic relationships.
